# Drug Combination Recommendation Model for Systemic Lupus Erythematosus and Antiphospholipid Syndrome

**DOI:** 10.3390/ph18081224

**Published:** 2025-08-19

**Authors:** Ling Wang, Zhengyang Zhang, Ziheng Zhang, Tie Hua Zhou, Keun Ho Ryu

**Affiliations:** 1 Department of Computer Science and Technology, School of Computer Science, Northeast Electric Power University, Jilin City 132013, China; smile2867ling@neepu.edu.cn (L.W.); 2202301030@neepu.edu.cn (Z.Z.); 2202400833@neepu.edu.cn (Z.Z.); 2 Data Science Laboratory, Faculty of Information Technology, Ton Duc Thang University, Ho Chi Minh City 700000, Vietnam; khryu@tdtu.edu.vn; 3 Biomedical Engineering Institute, Chiang Mai University, Chiang Mai 50200, Thailand; 4 Department of Computer Science, College of Electrical and Computer Engineering, Chungbuk National University, Cheongju 28644, Republic of Korea

**Keywords:** drug recommendations, drug combination frequency, risk assessment, genetic interaction information, drug interactions

## Abstract

**Background**: Systemic Lupus Erythematosus (SLE) and Antiphospholipid Syndrome (APS) are two common autoimmune disorders for which the choice of drug regimen is clinically crucial. However, due to drug-drug interactions and individual differences, the therapeutic process faces greater risks. **Methods**: In this study, we propose a drug recommendation model that combines drug combination frequency, risk assessment, and genetic interaction information with the aim of providing personalized, low-risk treatment options for patients with lupus erythematosus and antiphospholipid syndrome. We extracted drug combination frequencies and drug-gene interaction information from data sources, such as the MIMIC-III clinical database, Drug Bank, and Gene Expression Omnibus. The model comprehensively evaluates the frequency of drug combinations, the risk level, and the gene interaction information through a greedy algorithm to recommend the optimal drug alternatives. **Results**: The experimental results show that the model is able to effectively reduce the potential risk between drugs while ensuring the drug treatment effect. In addition, the performance evaluation of the drug recommendation model shows that the model performs well under different drug combinations and clinical scenarios, and can provide clinicians with effective drug substitution suggestions. **Conclusions**: This study provides an important theoretical basis and technical support for advancing the realization of personalized therapy and precision medicine.

## 1. Introduction

Systemic Lupus Erythematosus (SLE) and Antiphospholipid Syndrome (APS) are two common autoimmune disorders with complex clinical manifestations and diverse therapeutic needs [1,2]. Systemic Lupus Erythematosus is characterized by chronic inflammation involving multiple organ systems, including the skin, kidneys, heart, and nervous system, and is often accompanied by abnormal activation of the immune system. Antiphospholipid syndrome, on the other hand, is an autoimmune disease characterized by recurrent thrombosis, which may lead to serious complications such as stroke, deep vein thrombosis, and miscarriage. The treatment of these diseases is challenging, as the choice of drugs is closely related to individual patient differences [3], and many drugs may cause drug-drug interactions or adverse reactions, thus requiring more precise and personalized treatment regimens [4].

SLE and APS are both autoimmune diseases, and APS always co-occurs with SLE, or is considered a stage or complication in the core development of SLE, because long-term use of immunosuppressants and anticoagulants could lead to serious health risks due to interactions and side effects. Specifically, SLE patients often have antiphospholipid antibodies, which could lead to thrombosis, a specific manifestation of APS. APS patients also often have a thrombosis risk; some APS patients will develop into SLE patients as the disease progresses. Due to the similarities in clinical manifestations and immune mechanisms, it required personalized drug treatment recommendations to address the diverse symptoms and complex drug reactions.

With the continuous development of precision medicine and personalized treatment concepts, drug recommendation models have become an important tool in clinical treatment [5]. The goal of the drug recommendation model is to provide patients with the most appropriate treatment plan based on their clinical data, drug properties, drug interactions, and genetic information [6]. Traditional drug selection often relies on clinical experience and drug guidelines; however, this approach is not always optimal in complex diseases and multiple dosing situations [7]. Drug-drug interactions and individual differences may lead to serious adverse effects [8], which are particularly risky when using drugs in patients with systemic lupus erythematosus and antiphospholipid syndromes, among others. Therefore, data-driven drug recommendation models can provide clinicians with a scientific basis for optimizing drug regimens [9].

In recent years, research on drug recommendation models has gradually gained attention, especially models that combine data from various aspects, such as frequency of drug combinations, risk assessment, and genetic interaction information. Analysis of the frequency of drug combinations [10] can reveal common patterns of drug use in specific diseases, while risk assessment [11] helps to identify potential adverse drug reactions and avoid the use of high-risk drugs. Gene interaction information [12], on the other hand, provides a more personalized basis for drug recommendation, and by analyzing the relationship between a drug and a patient’s genes, it is possible to further optimize treatment regimens and avoid adverse drug reactions due to genetic differences [13].

The goal of this study is to construct a drug recommendation model based on the frequency of drug combinations, risk assessment, and gene interaction information, specifically for two diseases, systemic lupus erythematosus and antiphospholipid syndrome. We utilized data sources such as the MIMIC-III clinical database, DrugBank, and Gene Expression Omnibus to combine the frequency of drug combinations, risk level, and gene interaction information to perform drug recommendation by a greedy algorithm [14]. The innovation of the model is that it integrates the frequency of drug use, risk assessment, and genetic background information to provide patients with personalized drug alternatives from multiple dimensions [15]. By evaluating the performance of the recommendation model, we verified the effectiveness of the model in clinical applications and provided new ideas for the future development of personalized drug therapy [16].

## 2. Related Work

With the increasing availability of data in the medical field, drug recommendation models have become an important tool to enhance clinical decision support. These models provide personalized drug recommendations to individuals by combining different data sources, such as chemical structures of drugs, clinical trial results, and genetic data. However, the diversity of drugs and the complex interactions between drugs and genes make traditional drug recommendation methods face many challenges. In recent years, recommendation methods based on drug similarity, drug-gene interaction information, and risk assessment have gradually gained the attention of researchers and become an important development direction in the field of drug recommendation.

### 2.1. Drug Recommendation Model

Drug recommendation models provide individuals with personalized drug choices by integrating various characteristics of drugs. With the continuous advancement of data mining techniques and machine learning methods, drug recommendation models have become an important tool to enhance clinical decision support. In recent years, researchers have worked to improve traditional drug recommendation methods through more sophisticated algorithms and data sources.

Liu et al. [17] proposed LEADER, a large language model (LLM)-based drug recommendation method, by creating cue templates that enable the LLM to recommend drugs effectively. To solve the out-of-corpus problem of LLM in drug recommendation, the researchers adapted it with a new output layer and a tuning loss function. Considering the high computational cost of LLM reasoning, the authors introduce feature-level knowledge distillation techniques to transfer LLM capabilities to a more compact model. Experimental results show that the model is both efficient and effective. In contrast, our model not only integrates drug combination frequency and risk assessment when recommending drugs, but also incorporates gene interaction information, thereby providing more personalized and precise treatment plans. Tan et al. [18] proposed a symptom-based set-to-set small and safe drug recommendation framework (4SDrug). The approach ensures that the recommended drug set is both small and safe by designing a set representation with a similarity measure that combines symptom set aggregation and drug set enhancement. Experimental results show that 4SDrug exhibits significant enhancement on the MIMIC-III and NELL datasets, with the smallest recommended drug set and a 26.83% reduction in drug-drug interactions. Therefore, our model can not only provide safer drug alternatives, but also optimize treatment options based on the patient’s genetic background and disease characteristics, thereby reducing adverse reactions caused by genetic differences. Ye et al. [19] proposed a drug-target interaction prediction framework (KGE_NFM) that combines a knowledge graph (KG) and a recommender system. The framework integrates multimodal information through low-dimensional representation and a neural factorization machine (NFM), and achieves excellent prediction results on four benchmark datasets, especially in protein cold-start scenarios. Therefore, KGE_NFM is more suitable for recommendations at the drug-target level, while our proposed model further improves its adaptability to individual patient differences by combining drug combination frequency, risk assessment, and gene interaction information, especially in the treatment of complex autoimmune diseases such as SLE and APS.

### 2.2. Drug Risk Assessment

Drug risk assessment refers to the evaluation of potential risks during drug use by analyzing the interactions, including factors such as side effects, drug interactions, and individual differences. With the advancement of genomics, medicinal chemistry, and clinical data analysis technologies, drug risk assessment methods based on big data and artificial intelligence are gradually becoming a hot research topic.

Charoo et al. [20] discussed the potential risks of nitrosamines in pharmaceutical products and proposed strategies to control the risks. Studies have shown that nitrosamines or their precursors may be present in any formulation ingredient, and that high-risk ingredients should be avoided during manufacturing and measures taken to ensure that nitrosamine levels are below acceptable levels in the absence of suitable alternatives. Manufacturers should conduct in-depth studies on the production pathways of excipients and prevent the production of nitrosamine in combination with anti-nitrification inhibitors such as vitamin C. Lee et al. [21] provided an overview of the assessment of drug-drug interactions (DDIs), focusing on the role of cytochrome P450 (CYP) enzymes in drug metabolism and their impact on DDIs. Studies have shown that CYP enzymes are responsible for the biotransformation of a wide range of drugs and thus play a key role in drug metabolism and DDI.

### 2.3. Drug Substitution Recommendation

The purpose of the drug substitution recommendation model is to be able to quickly provide a suitable alternative drug in case of side effects or patient allergy to a drug. Traditional drug substitution recommendation methods mainly rely on the similarity calculation of drugs, however, this method usually ignores the complex interactions between drugs and the influence of genes. Therefore, in recent years, more and more studies have attempted to combine the genetic information of drugs, the side effect data of drugs, etc., to recommend more accurate drug substitution programs for individuals.

Marzolini et al. [22] reviewed the risk of drug-drug interactions (DDI) between nirmatrelvir/ritonavir (Paxlovid) and other drugs. Since ritonavir increases plasma concentrations of nirmatrelvir by inhibiting the CYP3A4 enzyme, combining it with other CYP3A4-metabolized drugs may lead to DDIs. The article suggests managing these risks by suspending the combined medication or by drug substitution, and provides a list of possible alternative medications to minimize the harms associated with DDIs. Gunn et al. [23] reviewed the literature on alcohol and cannabis sharing and explored whether cannabis is used as a substitute for alcohol or as a supplement. The research suggests that cannabis may substitute for the effects of alcohol in some populations, while it may enhance the effects of alcohol in others. The article analyzes multiple mechanisms, including patterns of sharing, timing of use, pharmacological interactions, and user characteristics, that may influence the substitution or complementary effects of cannabis.

In summary, research on drug recommendation, risk assessment, and alternative recommendations continues to evolve with advances in data science and artificial intelligence technologies. Drug recommendation models provide personalized treatment plans and reduce the risk of side effects by analyzing drug similarity, patient clinical data, and genetic information. Meanwhile, drug risk assessment combines drug-gene interactions to accurately assess drug side effects and interactions. The drug substitution recommendation model provides safe alternatives to avoid potential risks when drugs are not applicable. This paper combines drug similarity, genetic information and drug combination frequency to propose a recommendation strategy with integrated features, which promotes personalization and safety of drug therapy.

## 3. Results

### 3.1. Dataset

The data sources used in this paper include several authoritative biomedical databases, covering multiple dimensions such as clinical, genomic, and drug information. The patient’s hospitalized electronic medical record data were obtained from the MIMIC-III Clinical Database v1.4 [24], which contains detailed clinical information such as diagnostic records, diagnostic codes, and medications of real hospitalized patients. Second, the variant gene information was obtained from the GEO (Gene Expression Omnibus) database [25], which contains the gene expression profiles of individuals with systemic lupus erythematosus (SLE) and antiphospholipid syndrome (APS) and normal controls, and is used to mine the differentially expressed genes associated with the diseases and the potential molecular mechanisms. We use 80% of the dataset for training and 20% for testing.

For drug-related information, the DrugBank drug database [26] was used in this paper to obtain information on standard therapeutic drugs, drug classifications, and their targets for SLE and APS. Meanwhile, in order to investigate the potential risks or synergistic effects that may be associated with the combination of drugs, the drug interaction information was mainly obtained from the interaction databases DrugBank and DDInter [27] to further explore the patterns of combination use between disease-related drugs and the possible interactions of drug combinations. Table 1 shows the specific use descriptions.

As can be seen from the table, 6520 patients were screened from electronic medical record data, and 5193 genes were utilized for comparison. Therapeutic drugs were screened from the DrugBank database for approximately 204 drugs with approximately 1985 potential interactions.

### 3.2. Data Preprocessing

First, data sources consisted primarily of the DIAGNOSES_ICD and PRESCRIPTIONS tables in the MIMIC-III database. We constructed a cohort of immune-related individuals by screening individual patients for ICD-9 codes 7100 (SLE) and 28981 (APS). For each patient, their hospitalization records were associated with SUBJECT_ID and HADM_ID and all medication data were extracted. This also ensured that the dataset only contained information on individuals associated with lupus erythematosus and antiphospholipid syndrome, avoiding other diseases from interfering with the results.

Second, to ensure the uniqueness of the drug set and the cleanliness of the data, the drug records during each hospitalization were de-duplicated. By removing duplicate drug information, we formed a unique set of drugs for each hospitalization. In the drug combination construction phase, we generated all possible 1–4 tuples (i.e., combinations containing 1–4 drugs). For each drug combination, we counted its frequency of occurrence across all patients. By analyzing the frequency of drug combinations, we further filtered the drug combinations with no less than four occurrences, ensuring that the recommendation model is trained on common and effective drug combinations.

**Figure 1:**
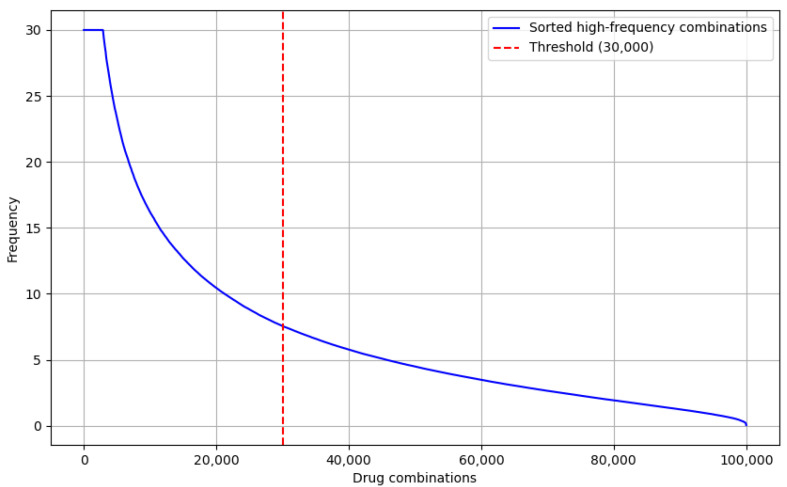
Drug-drug combinations frequency distribution.

Finally, to optimize the input data for the drug recommendation model, we sorted the drug combinations by frequency of occurrence and selected the top 30,000 high-frequency drug combinations, as shown in Figure 1. This filtering ensured that the recommendation model would focus on commonly used and effective drug combinations in real-world applications, avoiding the influence of low-frequency drug combinations on the results.

As shown in Figure 1, the trend of the drug combination frequency curve is clearly decaying; the frequencies are relatively turning to smooth lower when the drug combinations are huge. Especially from the 30,000 drug combinations, the frequency drops significantly, and the subsequent combinations are almost all low-frequency combinations. This distribution indicates that the top 30,000 high-frequency combinations could cover the majority of common drug combinations and shows that it is quite representative.

### 3.3. Drug Risk Level Calculation

During the construction of drug combinations, we generated different drug combinations for each patient’s drug use record and filtered combinations with a frequency below a certain threshold, retaining the more common drug combinations. The risk assessment between drugs was calculated by the above formula, and the drug interaction risk information was labeled. We then looked up the corresponding risk levels in the database based on the standardized names of the drug pairs and compared the risk levels for each drug combination. The risk information for each drug pair is categorized according to predefined rules.

From these, we selected several drug combinations with high frequency, and then calculated interaction strength scores for each of them, then differentiated the strengths according to our own evaluation scale and compared them with the DDInter drug database. As shown in Table 2:

Table 2 shows the calculated drug-drug interaction intensity scores and risk assessment level results. The factor score is calculated by drug combination and recommends frequency, potential interaction risk, and gene target overlapping. Risk levels are categorized as low (L), medium (M), and high (H) according to the drug-drug interaction intensity scores classification. The DDInter database also contains some of the existing drug combination risk levels annotated, which are based on clinical experience and testing. The comparison results show that most of the calculation results are consistent with the existing annotation levels, and our calculated results are closer to the real clinical decision-making results because of the combination with the real clinical diagnostic records, gene-target dataset, and drug-drug interaction calculations.

In addition, the statistical results of the risk level distribution reveal the distribution of drug combinations under different risk levels, as shown in Table 3.

Table 3 shows the drug-drug combinations distribution status for the different risk levels. According to the statistical results, the vast majority of drug combinations were labeled as moderate risk, with 21,829 combinations, the highest percentage. This means that most of the drug combinations pose some risk in clinical practice, but do not pose a serious health threat. In contrast, the second highest proportion of drug combinations with unknown risk was 6127, which may indicate that the risk of these combinations has not been adequately studied or cannot be clearly assessed in the available data.

The relatively small number of 1840 high-risk drug combinations suggests that there are a certain number of high-risk drug combinations among the drug combinations studied that require special attention and management. In contrast, the number of low-risk drug combinations was 107, suggesting that a small number of drug combinations have a low risk and may be safer choices for clinical use. Finally, the number of drug combinations with no risk was 97, indicating that the majority of drug combinations are multi-drug combinations with relatively few single-drug situations.

### 3.4. Drug-Gene Interaction Information

The core purpose of constructing a drug-gene network graph is to provide data support for subsequent greedy recommendation algorithms. By visualizing the interactions between drugs and genes and the risk levels of drugs, the potential effects of drug combinations and their interrelationships can be intuitively revealed. The pseudo-code for its greedy recommendation algorithm is presented in Algorithm A1 in Appendix A.

**Figure 2:**
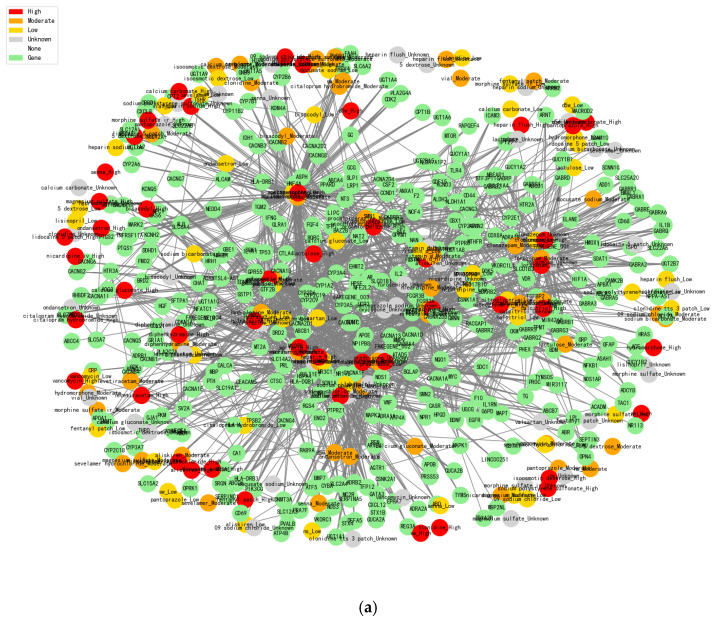

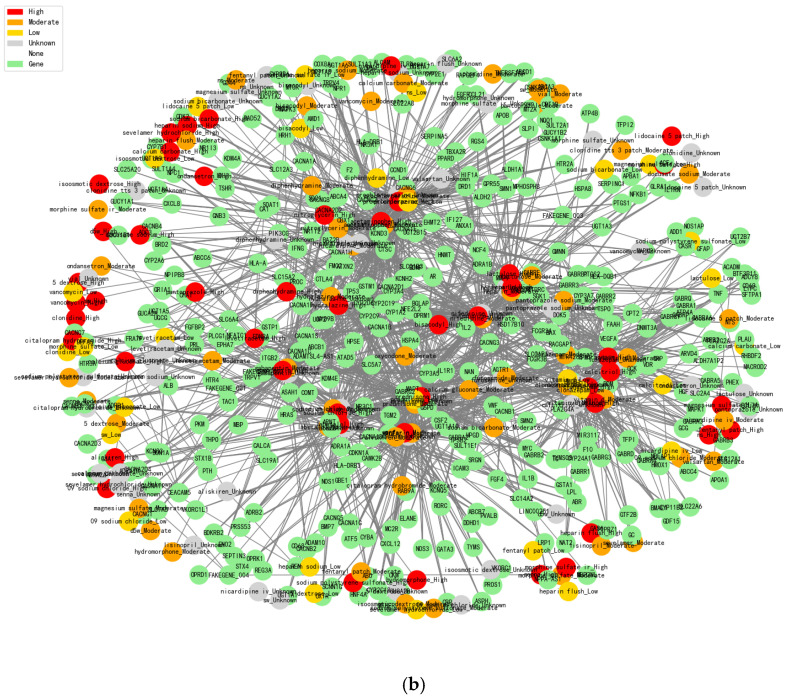
(**a**) shows the results of the analysis in the standard database. (**b**) shows our analysis based on our own data.

The construction of drug-gene network diagrams allows us to clearly present the risk level of drug combinations. As shown in Figure 2, the interaction information between drugs and genes can be displayed more intuitively. First, high-risk drug combinations are marked by the red color, and researchers can quickly identify potential high-risk combinations by color changes. Orange color indicates medium risk, yellow color indicates low risk, green color represents gene nodes, and gray represents unknown drug effect information. In addition, we use white to represent nodes with no effect information. Through the analysis of drug-gene interactions, the network diagram reveals potential substitution relationships between drugs. Drugs with overlapping gene intersections may have similar therapeutic mechanisms and, therefore, can be considered as mutual substitutes. In addition, the number of nodes in the two graphs has been presented in Table 4.

After calculating statistics between the GEO database (mutation genes information) and 6520 patients’ EMR to obtain the 5193 related genes, we then summarized the drug-related genes information together to get the final 375 shared genes, which involve 204 drugs (Figure 2a) and 187 drugs (Figure 2b). These selected genes are generally involved in immune system regulation, inflammatory responses, cytokine release, etc. Depending on the risk level of these drug combinations, the edges between drugs and genes denote the level of risk of the combination, which are 1717 and 1985, respectively. In addition, constructing this network graph helped us to identify a number of drug-gene interactions that may have been under-attended in existing studies. Potential drug-gene interactions may have an impact on efficacy or side effects, and this information can be further explored and validated through the presentation of the graph. By identifying these potential interactions, some unknown risks can be avoided in the selection of drug combinations, thus improving the clinical safety of drug combinations.

Figure 2a shows the results of a drug-gene interaction network analysis based on standard databases (such as MIMIC-III and DrugBank). Figure 2b is our constructed dataset with more detailed correlation calculations together, which is combined with real clinical cases’ drug combinations. Normal analysis is conducted without high-risk drug combination discovery as potential risks based on the standard databases, and our proposed model integrates personalized genetic information and drug interaction data, which could effectively reduce this kind of risk and more accurately recommend lower-risk drug alternatives in order to improve the drug therapy safety and efficacy.

Moreover, we collected the latest research papers’ drug combination information and real clinical cases, which contain the detailed drugs’ effect evaluation with quantitative indicators’ calculation, especially for the drug-gene interaction information and drug combination usage risk evaluation that makes our proposed model (Figure 2b) have more advantages than the usual statistical analysis (Figure 2a). It could enable the discovery of potential drug risks that were not fully annotated in the normal standard databases.

### 3.5. Drug Combination Recommendation

To further optimize the recommendation effect, we propose a greedy recommendation method for recommending suitable alternative drugs for users based on the characteristics of the input drug combinations (GRDC model). In the recommendation process, we consider three factors: the frequency of drug combinations, the risk assessment among drugs, and the genetic overlap. Specifically, we calculated an overall score for each drug candidate, which was a weighted combination of frequency score, risk penalty, and genetic overlap. Drugs with higher scores were considered better alternatives.

We have recommended suitable alternatives for high-risk drugs through the greedy recommendation method. The details of the high-risk drugs and their alternative recommendations are shown in Table 5. Where H = High (high risk), M = Moderate (moderate risk), and L = Low (low risk). First, the Risk Drug column shows the name of the risky drug, e.g., azathioprine, which is the target drug for which we need to find an alternative. Next, the Recommended Substitute column shows the alternative drug that is recommended for that high-risk drug, and the recommended alternative drug in the table is gabapentin.

To ensure the rationality of the recommendations, we assessed the substitutability of the drugs by calculating the similarity between them through the number of drugs acting on the same gene. The Similarity column demonstrates the similarity between the recommended drug and the target drug, with a value of 0.71, indicating that there is some similarity in the genes and the therapeutic mechanism of these two drugs. The Shared Genes Count column further reveals the degree of overlap between these two drugs at the gene level, indicating that there are seven identical genes of action between azathioprine and gabapentin.

The suitability of the recommended medications is also reflected in the composite score, with the Score column demonstrating the total score of the alternative medications. The score is a normalized result with a numerical range of 0–100. A higher score indicates a higher suitability of the recommended drug as an alternative drug. This score is calculated by taking into account a combination of drug frequency, risk class, and genetic overlap. A score of 78 indicates that gabapentin is a more suitable alternative drug. Further, the Original Risk column indicates that azathioprine is a medium-risk drug, while the Substitute Risk column indicates that the recommended alternative drug, gabapentin, has a low-risk rating.

Next, we evaluated the performance of the drug recommendation model and used several standard recommendation model evaluation metrics to measure its effectiveness, as shown in Table 6. First, we assessed the accuracy of the recommendation model by using the Precision@10 metric, which resulted in a result of 0.926, implying that 92.6% of the top 10 recommended drugs were actually relevant. Next, we looked at Recall@10, which measures the coverage of actually relevant drugs in the top 10 recommendations. Our result of 0.911 indicates that the model is able to recommend 91.1% of relevant drugs from all actually relevant drugs. F1@10, which is the reconciled average of Precision and Recall, is used to comprehensively assess the accuracy and recall of the recommendation model, and the result of 0.905 indicates that the recommendation model achieves good results in balancing precision and recall. Finally, the Hit@10 metric shows the proportion of the top 10 recommendations in which at least one relevant drug is recommended. Our result of 0.903 indicates that the model is able to provide at least one relevant drug in most cases. The pseudo-code for the recommended model evaluation criteria is shown in Algorithm A2 in Appendix A.

In order to further test the recommendation performance, LEADER, 4SDrug, and KGE_NFM were used as benchmarks to compare with our proposed GRDC model in Table 6. Our model’s superior performance on these metrics stems primarily from its comprehensive consideration of multiple factors, including drug combination frequency, gene interactions, and drug risk, which leads the drug recommendation accuracy rate to be higher, especially for discovering the potential effective drug combinations. By introducing a risk penalty mechanism, it would further improve the drug recommendation precision and recall.

### 3.6. Dataset Test Comparison

To further validate the robustness and generalizability of the proposed drug recommendation model, we applied the model to compare with the MIMIC-IV dataset [28]. The MIMIC-IV dataset not only contains a wider timeframe, but also has more comprehensive structured records, making it a more valuable reference dataset to evaluate the model performance in real-world clinical settings.

The performance comparison results between MIMIC-III and MIMIC-IV for the Top-10 recommendation tasks are shown in Table 7:

Compared with the MIMIC-III results, the recommendation model also expressed good performance on the MIMIC-IV dataset, which demonstrated good generalization across diverse patient compositions and time spans.

### 3.7. 3D Visualization

**Figure 3:**
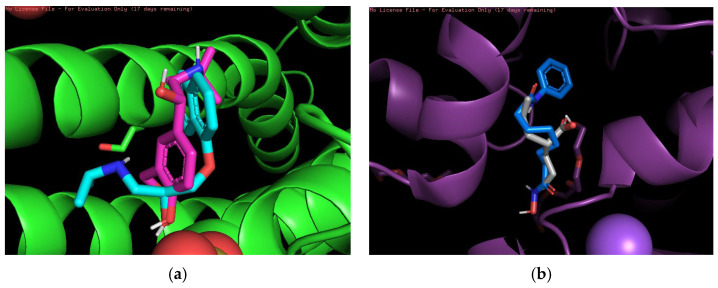
(**a**) Propranolol and Salbutamol. (**b**) Valproic Acid and Vorinostat.

Finally, 3D visualization was performed for each risky drug combination, which allows visualization of the drug permutations in terms of molecular structure. First, for the high-risk combinations, we used two groups of drugs, Propranolol and Salbutamol and Valproic Acid and Vorinostat, for visualization, as shown in Figure 3.

Figure 3a demonstrates the overlapping binding conformations of Propranolol (magenta) and Salbutamol (cyan blue) at the same target site locus. Both show some conformational differences in the hydrophobic pocket, indicating that they interact with the protein in a similar but not identical manner. The green helical structure is the α-helical region of the target protein. Furthermore, the combination shown in the figure may also indicate an overreaction of the cardiovascular system in certain circumstances. Figure 3b shows the conformational overlap of Valproic Acid (gray) with Vorinostat (blue) at the protein binding site. Both form interactions with the internal helical region of the protein (purple color), coordinating to the metal ion (purple ball) via carboxylic acid or amide groups, suggesting that they have a potential competitive binding mechanism and may have unintended effects on treatment.

**Figure 4:**
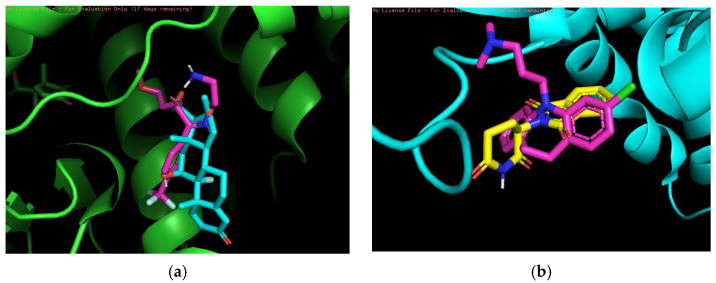
(**a**) Betamethasone and Fluvoxamine. (**b**) Clomipramine and Thalidomide.

In the medium-risk combination, we used two drugs, Betamethasone and Fluvoxamine and Clomipramine and Thalidomide, as shown in Figure 4. The overlapping binding conformations of Betamethasone (lime green) and Fluvoxamine (magenta) in the target proteins are shown in Figure 4a. Both have better spatial complementarity at the binding site, where Betamethasone shows a deeper embedding pattern, indicating that it forms stronger hydrophobic interactions in the hydrophobic pocket. The combination of these two drugs has clinical potential, especially in the setting of coexisting immune and depressive disorders. They act on different biological mechanisms (immune response and neurotransmitter systems) and could be used together in certain patient populations. Figure 4b shows the conformational overlap between Clomipramine (yellow) and Thalidomide (pink) at the same protein binding site. Both core structures form a tighter overlap, suggesting that they compete for the same binding site. The blue-green color of the protein secondary structure shows that both are located in regions where hydrogen bonding or π-π stacking may exist, thus enhancing the affinity of the ligand for the target. The combination of clomipramine and thalidomide may be used clinically in patients with immune system diseases and coexisting depression. The immunosuppressive effect of thalidomide may affect the metabolism of clomipramine, and vice versa.

**Figure 5:**
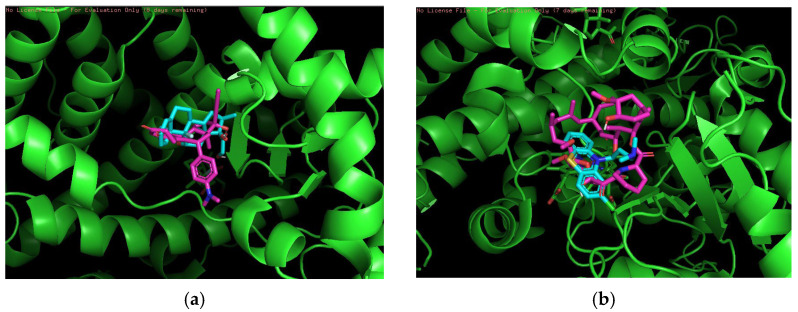
(**a**) Dexamethasone and Mifepristone. (**b**) Chlorpromazine and Tacrolimus.

Finally, two drug combinations, Dexamethasone and Mifepristone and Chlorpromazine and Tacrolimus, were used in low-risk combinations as shown in Figure 5. Figure 5a demonstrates a comparison of the binding conformations of Dexamethasone (cyan) and Mifepristone (magenta) inside the target protein. Both show highly complementary chimerism in the hydrophobic binding pocket, with the steroidal backbone of Dexamethasone forming an overlap with the aromatic ring system of Mifepristone, suggesting that they modulate target function in an antagonistic or synergistic manner. Dexamethasone can effectively control inflammation through its immunosuppressive effects, while mifepristone may modify the effects of dexamethasone by antagonizing glucocorticoids, especially during long-term use. The binding postures of Chlorpromazine (pink) and Tacrolimus (cyan) at the protein targets are illustrated in Figure 5b. Both form multiple spatial overlaps in the binding pocket involving different interaction modes, despite significant structural differences. The green bottom protein conformation exhibits a highly convoluted helical region, providing a complex three-dimensional space for ligand binding. The combination of chlorpromazine and tacrolimus may have clinical applications, particularly in the treatment of transplant patients or those on immunosuppressive therapy, where depression may be a problem. Chlorpromazine alleviates psychiatric symptoms by affecting the effects of neurotransmitters, while tacrolimus prevents rejection through its immunosuppressive effects.

## 4. Discussion

The drug recommendation model based on drug combination frequency, risk assessment, and gene interaction information proposed in this study has important clinical application potential, especially in the personalized treatment of individuals with systemic lupus erythematosus (SLE) and antiphospholipid syndrome (APS). By introducing drug frequency, risk assessment, and gene interaction information, the model not only improves the science and safety of drug recommendations, but also is able to recommend the most appropriate drug combinations according to the patient’s specific situation and reduce the occurrence of adverse reactions.

In clinical practice, individuals with SLE and APS often require long-term use of immunosuppressive and anticoagulant medications, and the interactions and side effects of these medications can lead to serious health risks. Therefore, the safety and efficacy assessment of drugs is particularly important. The risk assessment mechanism in this study can provide physicians with safer drug choices and avoid the use of high-risk drug combinations, thus effectively reducing the incidence of adverse drug reactions.

In addition, the interaction between drugs and genes is an important component of personalized medicine. With the continuous development of genomics and precision medicine, the relationship between drugs and patients’ genetic characteristics has become increasingly important. Drug recommendations based on genetic interaction information can provide patients with more biologically rational treatment plans and reduce adverse reactions and drug tolerance problems caused by individual differences.

In this study, although the model performed well for common drug combinations and standard genetic profiles, it may face challenges with rare drug combinations and atypical genotypes. Due to the training data sparseness for rare drug combinations, it also would lead to inaccurate recommendation results. To address this issue, future work will introduce an extended dataset by using the transfer learning method to improve the recommendation capabilities for rare combinations. For atypical genetic expressions, expanding genomic data and incorporating a personalized recommendation mechanism could help to improve the model scalability.

In order to further improve the treatment precision and wide suitability, the future work is focused on expanding data resources, particularly by integrating with clinical data from different regions and medical institutions, as well as multicenter clinical trial data. Moreover, patient feedback and follow-up data could help to facilitate dynamic adjustment and optimize the drug recommendation system in order to enhance its wide applicability. Furthermore, integrating more comprehensive genomic data will provide more precise support for personalized treatment, which is better for promotion and application in autoimmune disease treatment, especially for systemic lupus erythematosus (SLE) and antiphospholipid syndrome (APS). In summary, the drug recommendation model proposed in this study provides innovative ideas for the treatment of SLE and APS and demonstrates the potential of combining drug combination frequency, risk assessment, and genetic interaction information. In the future, with the continuous accumulation of clinical data and the optimization of algorithms, the drug recommendation model will play an even more important role in personalized medicine and precision therapy.

## 5. Materials and Methods

### 5.1. Motivation

Systemic Lupus Erythematosus (SLE) and Antiphospholipid Syndrome (APS) are complex autoimmune diseases that often require multi-drug combination therapy. However, existing clinical drug regimens rely on empirical or single-disease guidelines, and lack a systematic and quantifiable method for recommending drug combinations, which makes it difficult to effectively address the efficacy tradeoffs and interaction risks of multidrug combinations. In this study, based on the MIMIC-III clinical database, we extracted real medication data, constructed a recommendation model integrating medication frequency and risk assessment, and introduced gene-drug mapping to enhance the biological explanatory power by combining disease target information. This method provides data-driven decision support for individualized combinations of SLE and APS, which is expected to improve the therapeutic efficacy and drug safety.

**Figure 6:**
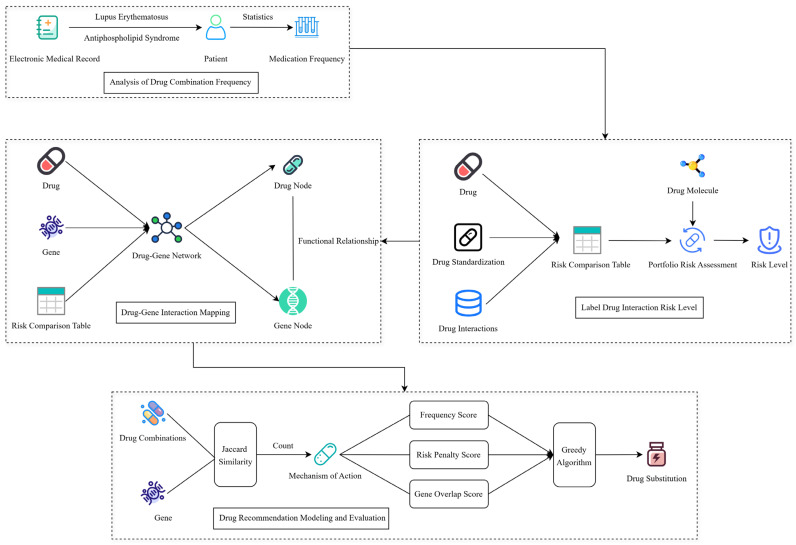
Drug combination substitution model and framework.

As shown in Figure 6, extracting the patients’ combined drug use medication frequency based on MIMIC-III datasets (Section 5.2) and calculating the drug-drug interaction risk levels (Section 5.3) based on the collected drugs’ detailed information. Then, combining with frequency, risk levels, and biological target information to construct a drug-gene interaction network (Section 5.4), and finally implementing the low-risk drug combination recommendation (Section 5.5) by our proposed Greedy Recommendation Drug Combination (GRDC) model. Ultimately, the greedy algorithm is introduced to generate individualized drug substitution and recommendation schemes by fusing frequency scores, risk penalties, and gene overlap scores.

### 5.2. Drug Combination Frequency Analysis

Comprehensive EMR records were gathered from MIMIC- III database, which were stored in DIAGNOSES_ICD table, and searched for information from the attribute SUBJECT_IDs volumes about ICD-9 code 7100 (Systemic Lupus Erythematosus) and ICD-9 code 28981 (Antiphospholipid Syndrome). Then, as one part of the self-constructed dataset, which includes this selected EMR information. Subsequently, we extracted all medication records of the above patients during their hospitalization (indexed by SUBJECT_ID with HADM_ID) using the PRESCRIPTIONS table. To ensure the uniqueness and cleanliness of the combination, the names of the medications involved during each hospitalization are de-duplicated to form a unique set of medications representing each hospitalization. Only retaining the patients’ information that repeated SLE or APS diagnosis codes across multiple hospitalizations, which could exclude samples introduced by miscoding or misdiagnosis during a single hospitalization. In addition, the patients’ medication use records were further cross-referenced to ensure the original dataset quality and enhance the diagnosis records’ confidence.

In the combination construction phase, we generated all possible 1–4 tuples (i.e., combinations of one to four drugs) for each drug set and counted their frequency of occurrence among all individuals. To filter out chance combinations, we retained only those drug combinations with a frequency of no less than four occurrences. The combinations were then sorted in descending order of frequency, and the top 30,000 high-frequency combinations were selected as inputs for the construction of the Drug Interaction Labeling and Recommendation (DILR) module.

### 5.3. Drug Interaction Risk Label

To assess the safety of drug combinations, the drug combinations of relevant individuals were subjected to drug interaction risk calculation. Meanwhile, to eliminate the effect of naming inconsistency, we introduced a drug name standardization strategy to compare with the standard names of drugs in the DrugBank database and to unify the format of the drug names in the combination file and the interaction table, including lowercase conversion, special character removal, and bracketed content elimination. Then, for each drug combination, all possible drug pairs were extracted. The corresponding interaction strengths were calculated separately according to the number of their combinations.

For drug combinations, we can make calculations in terms of molecular structure. Root Mean Square Deviation (RMSD) is used to assess the stability of the molecular docking results and the tightness of the binding. Measures the degree of structural deviation by calculating the difference in the atomic positions of a molecule in different binding conformations relative to a reference conformation (usually the lowest energy, optimal binding mode) [29]. The formula for RMSD is shown below.

(1) $$RMSD=\sqrt{\frac{1}{N}\sum_{i=1}^{N}{\left(x_{i}-x_{ref}\right)}^{2}}$$

- N: Represents the number of elements involved in the calculation.
- X_i_: Represents the position of the i-th atom in the target structure.
- X_ref_: Represents the position of the corresponding atom in the reference structure.

Furthermore, the drug-drug interaction risk score is based in part on the affinity and root mean square deviation (RMSD) metrics derived from molecular docking. We used AutoDockTools v1.5.6 software for molecular docking simulations. Drug molecules were normalized prior to docking, including removal of water molecules, addition of hydrogen atoms, and assignment of Gasteiger charges. The three-dimensional structure of the target protein was obtained from the PDB database, and a grid file for the receptor protein was generated using the AutoGrid tool. During the docking process, the optimal docking mode (mode 1) was obtained for each drug combination. Affinity represents the binding free energy (in kcal/mol), with lower values indicating more stable binding. RMSD measures the deviation between the predicted structure and the reference optimal conformation; generally, a smaller RMSD indicates more reliable docking results [30]. The equation for the calculation of affinity is shown below:

(2) $$∆G^{{}^{\circ}}=\left\langle U_{PL}\right\rangle -\left\langle U_{P}\right\rangle -\left\langle U_{L}\right\rangle +\left\langle W_{PL}\right\rangle -\left\langle W_{P}\right\rangle -\left\langle W_{L}\right\rangle -T∆S_{config}$$

- ⟨U⟩: The potential energy of the system, calculated first for the protein-ligand complex, and then for the protein and ligand separately.
- ⟨W⟩: The work performed on the system, calculated through PB (Poisson-Boltzmann) or GB (Generalized Born) methods. It is the average work for the system and the corresponding components (protein, ligand).
- $∆S_{config}$: The change in configuration entropy, often related to the changes in the structure of the system during the complex formation.
- $\left\langle U_{PL}\right\rangle$: Average potential energy of the protein-ligand complex; $\left\langle U_{P}\right\rangle$: Average potential energy of the isolated protein; $\left\langle U_{L}\right\rangle$: Average potential energy of the isolated ligand; $\left\langle W_{PL}\right\rangle$: Solvation free energy of the protein-ligand complex; $\left\langle W_{P}\right\rangle$: Solvation free energy of the protein; $\left\langle W_{L}\right\rangle$: Solvation free energy of the ligand; **T**: System temperature, typically set at 298 K, used to evaluate the entropic contribution to binding free energy.

After calculating the results of RMSD and Affinity, the next step of scoring can be performed [31]:

(3) $${Score}_{mode}=\alpha \times (Normalized\;Affinity)+\beta \times (Inverse\;Normalized\;RMSD)$$

For comparison of affinities, the affinity values are normalized. The minimum affinity value is set to 0, and the maximum affinity value is set to 1. The formula for this is:

(4) $$Normalized\;Affinity=\frac{{Affinity}_{mode}-min\left(Affinity\right)}{max\left(Affinity\right)-min\left(Affinity\right)}$$

To emphasize the need for smaller RMSD, we normalize it similarly. The smallest RMSD is set to 0, and the largest to 1. The formula is:

(5) $$Inverse\;Normalized\;RMSD=1-\frac{{RMSD}_{mode}-min\left(RMSD\right)}{max\left(RMSD\right)-min\left(RMSD\right)}$$

The lower the free energy value, the stronger and more stable the binding of the mode. Therefore, the free energy is our primary reference standard when assessing the binding strength. Suppose we take the free energy as the basic scoring item and set its percentage weight as α. The RMSD value reflects the difference in spatial structure between a given mode and the optimal mode (usually MODE 1). In general, a smaller RMSD implies that the structure of the mode is more similar and tighter to the optimal mode, while a larger RMSD indicates a larger structural difference between the mode and the optimal mode. Therefore, we use the difference in RMSD as an auxiliary scoring term and set its weight as β, where β is smaller than α.

For two drugs, the combination score can be expressed by taking the average of the two drug scores:

(6) $$Overall\;Score=\frac{{Score}_{drug1}+{Score}_{drug2}}{2}$$

For all three drugs, their individual scores were calculated first:

(7) $$Avg\;Individual\;Score=\frac{S_{A}+S_{B}+S_{C}}{3}$$

where each $S_{X}=\alpha \times {Affinity}_{X}+\beta \times {NRMSD}_{X}$. Then, calculate the two-by-two mutual scores. First, calculate the interaction energies for AB, AC, and BC (the binding energies for each drug pair):

(8) $${Interaction\;Score}_{XY}=f\left({Affinity}_{XY},{RMSD}_{XY}\right)$$

Then, take the average:

(9) $${Avg\;Interaction\;Score}_{ABC}=\frac{{Interaction\;Score}_{AB}+{Interaction\;Score}_{AB}+{Interaction\;Score}_{BC}}{3}$$

Finally, the calculation of the composite score was carried out:

(10) $${Overall\;Score}_{ABC}=\gamma \times Avg\;Interaction\;Score+(1-\gamma )\times {Avg\;Interaction\;Score}_{ABC}$$

where γ∈[0,1], typically recommended values for γ=0.5.

For the four drugs, their individual scores were calculated first:

(11) $$Avg\;Individual\;Score=\frac{S_{A}+S_{B}+S_{C}+S_{D}}{4}$$

Then, calculate the interaction energy of AB, AC, AD, BC, BD, and CD:

(12) $${Interaction\;Score}_{ij}=f\left({Affinity}_{ij},{RMSD}_{ij}\right)$$

Ask for averages on them:

(13) $${Avg\;Individual\;Score}_{ABCD}=\frac{1}{6}\sum_{i<j}{Individual\;Score}_{ij}$$

A composite score is given at the end:

(14) $${Overall\;Score}_{ABCD}=\gamma \times Avg\;Interaction\;Score+(1-\gamma )\times {Avg\;Interaction\;Score}_{ABCD}$$

where γ∈[0,1], typically recommended values for γ=0.5.

Finally, we can categorize the scoring intervals into Strong, Medium, and Weak based on the calculated Overall Score. The division is as follows:

- High Interaction: When the Overall Score ≥0.75, indicating a strong interaction.
- Moderate Interaction: When 0.5≤ Overall Score <0.75, indicating a moderate interaction.
- Low Interaction: When the Overall Score <0.5, indicating a weak interaction.

For cases where there are multiple risk levels in the portfolio, the most severe interaction level is selected as the overall risk label for+ the portfolio, prioritized by High > Moderate > Low > Unknown.

### 5.4. Drug-Gene Interaction Network

In order to enhance the clinical interpretability of the drug combination recommendation model and to introduce mechanistic information at the target level, a Drug-Gene Interaction Network (DGIN), which integrates drug risk classes and target genes, is therefore constructed. The graph is structured as a heterogeneous network with two types of nodes: drug nodes and gene nodes, and the edges represent the action relationship between drugs and their target genes.

A line-by-line traversal of the drug combination files labeled with the interaction risk level was performed to resolve each set of drug combinations and their risk levels, and to normalize each of them. We modeled each drug as a network node in the form of “drug name_risk level” (e.g., hydroxychloroquine_High), ensuring that the same drug exists as a different node under different risks, and each drug node is edge-connected to its corresponding gene target.

The constructed network has the following characteristics:

- Node types: drug nodes (with risk information) and gene nodes.
- Edge type: interaction between drug and target (undirected edge representation).
- Color coding: drug node colors are labeled according to risk level (e.g., High = red, Moderate = orange, Low = gold, Unknown = gray, None = white), and gene nodes are uniform green.
- Structural features: the same drug may exist in multiple risk classes (multiple versions of the node), and multiple drugs may share gene nodes, forming a “star structure” or “cluster structure”.

### 5.5. Drug Recommendation Model and Evaluation

In order to assist clinical rational drug use and potential alternative drug screening, this paper constructs a drug recommendation model based on a greedy strategy that considers the frequency of drug combinations, the risk level of drug interactions, and the similarity of drug target genes. The model can predict and recommend new drugs with potentially similar mechanisms, but lower interaction risks based on known drug combinations, aiming to realize safer and biologically rational alternative recommendations. The recommendation model takes as input an existing drug combination (e.g., 1 or 2 drugs) and uses Jaccard similarity (target gene intersection size/concatenation size) to measure the mechanistic similarity between drugs. This method is a heuristic measurement calculation and fails to cover the directionality (agonist vs. antagonist), gene expression levels, and downstream pathway effects. Pre-existing drug combinations from the EHR were extracted, and high-risk drug combinations were replaced with low- to moderate-risk ones. Drug candidates were screened in the set of all occurring drugs, and their composite recommendation scores were calculated based on the following three score metrics:

- Frequency Score: Indicates the frequency of co-occurrence of the drug candidate with the current combination of drugs. All 2~3 tuples formed by combinations with input drugs are tabulated, and the number of occurrences of the combination in real clinical data is accumulated.
- Risk Penalty Score: Based on the drug pairs formed by the drug candidates and each drug in the current combination, their interaction levels (e.g., High, Moderate, Low, etc.) are looked up in the preconstructed risk level dictionary and accumulated based on a set penalty weight function (e.g., High: −10, Moderate: −5, Low: −1).
- Based on the target information recorded in the drug-gene network, the similarity between the drug candidate and the current combination drug at the target level is calculated. Specifically, by calculating the intersection count (Shared Target Count) with the set of target genes between each input drug and accumulating them.

The final recommendation score consists of a weighted sum of the above three components, with the following formula:

(15) Total Score=α×Frequency+β×Gene Overlap+γ×Risk Penalty

where the default weights are: α = 1.0, β = 2.0, γ = 1.0.

**Figure 7:**
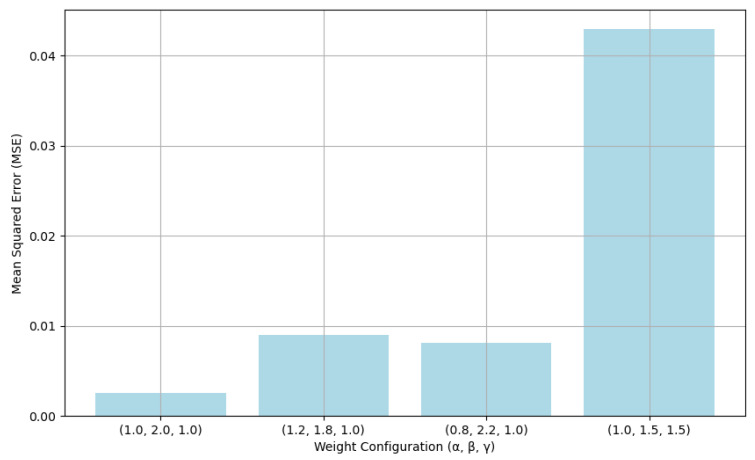
Different weight configurations on model performance impact.

As shown in Figure 7, weight combination selection for parameters α, β, γ is based on Mean Squared Error (MSE) values comparison for each weight configuration, which is a regression-based optimization process to test the impacts of different weight combinations for recommendation accuracy. In the testing, the weight configuration (α = 1.0, β = 2.0, γ = 1.0) is the best performance because of the smallest value of MSE and the default weight configuration is the best option for improving the recommendation performance.

## 6. Conclusions

This study proposes a drug recommendation model based on drug combination frequency, risk assessment, and gene interaction information, aiming to provide personalized drug treatment plans for individuals with Systemic Lupus Erythematosus (SLE) and Antiphospholipid Syndrome (APS). By analyzing the frequency of patients’ drug combinations and combining drug risk assessment with gene interaction information, a comprehensive recommendation model was constructed, which can effectively reduce the recommendation of high-risk drugs and improve the biological reasonableness of recommendations.

In this study, the proposed drug recommendation GRDC model involves the co-calculation with drug combination frequency, drug-drug interaction risk, and genetic interaction information in order to effectively recommend low-risk alternative drug combinations. Experimental results demonstrate that the GRDC model outperforms several existing representative methods (such as LEADER, 4SDrug, and KGE_NFM) in terms of precision and recall, even for drug combinations using safety and personalized recommendation capabilities. This model has strong scalability and potential usage for clinical application, particularly in personalized medication recommendations for systemic lupus erythematosus (SLE) and antiphospholipid syndrome (APS) disease treatments. Although preliminary results have been achieved in this study, further validation and optimization are needed. In order to improve the scalability and wide suitability of our proposed model, future work will focus on medical area knowledge base construction for extending more clinical data, and considering different domains, regions, and clinical settings, and updating new drugs and gene information. Exploring more effective algorithms and further improving the model’s accuracy and real-time performance.

## Figures and Tables

**Table 1:** Data Distribution.

Dataset Name	Number	Introduction
MIMIC-III Clinical Database	6520	Sampling of patients
Gene Expression Omnibus	5193	Genetic control
DrugBank	204	Therapeutic Drugs
DDInter	1985	Interaction

**Table 2:** Drug Combination Related Information Sheet (H = High, M = Moderate, L = Low, U = Unknow).

Drug Combination	Frequency	Score	Risk Level	DDInter
(Hydralazine, Labetalol)	30	0.65	M	M
(Labetalol, Prednisone)	29	0.54	M	M
(Hydralazine, Labetalol, Prednisone)	29	0.63	M	M
(Clonidine, Hydralazine, Prednisone)	24	0.85	H	M
(Clonidine, Prednisone)	24	0.46	L	M
(Pantoprazole, Prednisone)	24	0.54	M	U
(Hydralazine, Labetalol, Prednisone, Sodium Chloride)	23	0.73	M	M
(Dextrose, Clonidine, Prednisone)	12	0.43	L	M
(Clonidine, Heparin, Prednisone)	11	0.46	L	M
(Bisacodyl, Clonidine, Prednisone)	10	0.44	L	M

**Table 3:** Risk Level Distribution.

Drug Name	Quantities
Moderate	21,829
Unknown	6127
High	1840
Low	107
None	97

**Table 4:** Drug Gene Nodes and Edges.

Name	Ours Gene	Standard Gene
drug nodes	187	204
gene nodes	375	375
edges	1717	1985

**Table 5:** Recommended Drug Analysis (H = High, M = Moderate, L = Low).

Risk Drug	Recommended Substitute	Similarity	Genes	Score	Original Risk	Substitute Risk
azathioprine	gabapentin	0.71	7	78	M	L
prednisone	acetaminophen	0.78	12	90	H	M
warfarin	prednisone	0.70	6	75	M	L
pantoprazole	clonidine	0.66	5	72	M	L
cyclophosphamide	labetalol	0.75	10	85	H	M
sodium chloride	hydralazine	0.65	4	70	H	M
aspirin	labetalol	0.68	9	74	M	L
gabapentin	warfarin	0.63	3	68	L	L
hydroxychloroquine	nifedipine	0.82	15	95	H	L
belimumab	methotrexate	0.72	8	80	M	M

**Table 6:** Drug Recommendation Model Performance.

Method	Precision@10	Recall@10	F1@10	Hit@10
GRDC	92.6%	91.1%	90.5%	90.3%
LEADER [17]	89.3%	87.8%	88.5%	86.9%
4SDrug [18]	86.2%	84.1%	85.1%	83.7%
KGE_NFM [19]	84.5%	83.2%	83.8%	82.5%

**Table 7:** Dataset Test Indicators Under Different Datasets.

Dataste	Precision@10	Recall@10	F1@10	Hit@10
MIMIC-III	92.6%	91.1%	90.5%	90.3%
MIMIC-IV	91.2%	89.6%	90.1%	89.4%

## Data Availability

A publicly available dataset was analyzed in this study. The data can be found here: https://go.drugbank.com/ (accessed on 5 January 2024. Version: 6.0), https://ddinter.scbdd.com/ (accessed on 4 September 2020. Version: 1.0), https://physionet.org/content/mimiciii/1.4/ (accessed on 4 September 2016. Version: 1.4), https://www.ncbi.nlm.nih.gov/geo/ (accessed on 18 July 2020. Version: 2.0).

## Appendix A

Algorithm A1 recommends potential alternative drugs for each input drug combination (usually 1 or 2 drugs) through a greedy strategy. The recommendation process takes into account the historical frequency of drug combinations, the risk of drug-drug interactions, and the similarity of target genes. For each candidate drug, its frequency score, risk penalty score, and target gene overlap score with the current drug combination are calculated, and the top N drugs with the highest scores are ultimately recommended as alternative drugs by weighted score ranking.

**Algorithm A1** Drug Recommendation Model Algorithm for Greedy Strategies
**Require:** current_drugs, combo_freq_dict, risk_dict, gene_map, *α*, *β*, *N* **Ensure:** Top_N_Recommendations 1: **Begin** 2: Normalize current_drugs 3: all_drugs ← union of all drugs in combo_freq_dict 4: candidate_drugs ← all_drugs − current_drugs 5: scored_candidates ← empty list 6: **for** each drug_c in candidate_drugs **do** 7: extended_combo ← current_drugs ∪ {drug_c} 8: freq_score ← 0 9: **for** each subset in combinations of extended_combo with size 2 to 3 **do** 10: freq_score ← freq_score + combo_freq_dict.get(subset, 0) 11: **end for** 12: risk_score ← 0 13: **for** each drug_d in current_drugs **do** 14: pair ← {drug_c, drug_d} 15: risk ← risk_dict.get(pair, “Unknown”) 16: risk_score ← risk_score + penalty_value(risk) 17: **end for** 18: overlap_score ← 0 19: **for** each drug_d in current_drugs **do** 20: overlap_score ← overlap_score + size of (gene_map[drug_c] ∩ gene_map[drug_d]) 21: **end for** 22: total_score ← *α*× freq_score + *β* × overlap_score + risk_score 23: Append (drug_c, total_score) to scored_candidates 24: **end for** 25: Sort scored_candidates in descending order of total_score 26: Top_N_Recommendations ← top *N* elements from scored_candidates 27: **End**

**Table A1:** Algorithm A1—Drug Recommendation Model Algorithm.

Notation	Description
current_drugs	Set of drugs currently prescribed to the patient
all_drugs	All drugs found in the frequency dictionary
candidate_drugs	Drugs not in current_drugs, considered for recommendation
combo_freq_dict	Dictionary mapping drug combinations (2–3 drugs) to their usage frequency
risk_dict	Dictionary indicating risk score for drug pairs
gene_map	Mapping of each drug to its associated genes
α	Weight of frequency score in final recommendation
β	Weight of gene overlap score in final recommendation
freq_score	Frequency score of candidate drug with current drugs
risk_score	Aggregated risk penalty from candidate to current drugs
overlap_score	Number of overlapping genes between candidate and current drugs
total_score	Combined score: α × freq_score + β × overlap_score + risk_score
Top_N_Recommendations	Top-N drugs with highest total score

Algorithm A2 is used to evaluate the performance of drug recommendation models. The accuracy and effectiveness of the recommendation model are measured by randomly selecting some of the drugs from the real drug portfolio as known drugs and generating alternative drugs using the recommendation model, and then calculating metrics such as Precision, Recall, F1 and Hit. The evaluation process provides quantitative feedback on model performance by sampling multiple times and calculating the average value of each metric to help optimize recommendation effectiveness.

**Algorithm A2** Recommended Model Evaluation Algorithm
1: **Begin** 2: Initialize precision_list, recall_list, f1_list, hit_list as empty lists 3: tested ← 0 4: **for** each combination in combo_data **do** 5: **if** size of combination < 3 **then** 6: Skip the combination 7: **end if** 8: Randomly select 2 drugs from combination as known_drugs 9: true_drugs ← remaining drugs from combination 10: recommended ← GreedyDrugRecommendation(known_drugs, combo_freq_dict, risk_dict, drug_gene_map, top_k) 11: recommended_set ← set of recommended drugs 12: true_positives ← recommended_set ∩ true_drugs 13: precision ← |true_positives|/top_k 14: recall ← |true_positives|/|true_drugs| 15: **if** precision + recall > 0 **then** 16: f1 ← 2 × precision × recall/(precision + recall) 17: **else** 18: f1 ← 0 19: **end if** 20: hit ← 1 if true_positives not empty else 0 21: Append precision, recall, f1, hit to respective lists 22: tested ← tested + 1 23: **if** tested ≥ sample_size **then** 24: Break 25: **end if** 26: **end for** 27: Calculate mean(precision_list), mean(recall_list), mean(f1_list), mean(hit_list) 28: Output EvaluationMetrics as (mean precision, mean recall, mean f1, mean hit) 29: **End**

**Table A2:** Algorithm A2—Model Evaluation Algorithm.

Notation	Description
combo_data	Dataset of known multi-drug combinations
known_drugs	Randomly selected subset (usually 2 drugs) for input
true_drugs	Remaining drugs used as ground truth for evaluation
recommended	Drugs recommended by the model for given known_drugs
top_k	Number of drugs to recommend
precision@k	Proportion of recommended drugs that are correct (in true_drugs)
recall@k	Proportion of true drugs that are correctly recommended
f1@k	Harmonic mean of precision and recall
hit@k	Binary: whether at least one true drug was recommended (1 or 0)
sample_size	Total number of sampled test cases for evaluation
precision_list	List to store precision values across test cases
recall_list	List to store recall values
f1_list	List to store F1 scores
hit_list	List to store hit values (0 or 1)

## References

[1] Siegel C., Sammaritano L. (2024). Systemic lupus erythematosus: A review. JAMA.

[2] Knight J., Branch D., Ortel T. (2023). Antiphospholipid syndrome: Advances in diagnosis, pathogenesis, and management. BMJ.

[3] Ashton M. (2022). Individual Differences and Personality.

[4] Gatla T. (2024). An innovative study exploring revolutionizing healthcare with ai: Personalized medicine: Predictive diagnostic techniques and individualized treatment. Int. J. Adv. Res. Interdiscip. Sci. Endeav..

[5] Fountzilas E., Tsimberidou A., Vo H., Kurzrock R. (2022). Clinical trial design in the era of precision medicine. Genome Med..

[6] Ingram B. (2011). Clinical Case Formulations: Matching the Integrative Treatment Plan to the Client.

[7] Masnoon N., Shakib S., Kalisch-Ellett L., Caughey G. (2017). What is polypharmacy? A systematic review of definitions. BMC Geriatr..

[8] Duong T., Valeyrie-Allanore L., Wolkenstein P., Chosidow O. (2017). Severe cutaneous adverse reactions to drugs. Lancet.

[9] Holloway K., Bennett T., Farrington D. (2006). The effectiveness of drug treatment programs in reducing criminal behavior: A meta-analysis. Psicothema.

[10] Zwart-van Rijkom J., Uijtendaal E., Ten Berg M., Van Solinge W., Egberts A. (2009). Frequency and nature of drug–drug interactions in a Dutch university hospital. Br. J. Clin. Pharmacol..

[11] Covello V., Merkhofer M. (2013). Risk Assessment Methods: Approaches for Assessing Health and Environmental Risks.

[12] Sherman B., Hao M., Qiu J., Jiao X., Baseler M., Lane H., Imamichi T., Chang W. (2022). DAVID: A web server for functional enrichment analysis and functional annotation of gene lists (2021 update). Nucleic Acids Res..

[13] Zazzara M., Palmer K., Vetrano D., Carfì A., Onder G. (2021). Adverse drug reactions in older adults: A narrative review of the literature. Eur. Geriatr. Med..

[14] Zhao Z., Zhou M., Liu S. (2021). Iterated greedy algorithms for flow-shop scheduling problems: A tutorial. IEEE Trans. Autom. Sci. Eng..

[15] Colledge-Frisby S., Ottaviano S., Webb P., Grebely J., Wheeler A., Cunningham E., Hajarizadeh B., Leung J., Peacock A., Vickerman P. (2023). Global coverage of interventions to prevent and manage drug-related harms among people who inject drugs: A systematic review. Lancet Glob. Health.

[16] Wang M., Herbst R., Boshoff C. (2021). Toward personalized treatment approaches for non-small-cell lung cancer. Nat. Med..

[17] Liu Q., Wu X., Zhao X., Zhu Y., Zhang Z., Tian F., Zheng Y. (2024). Large language model distilling medication recommendation model. arXiv.

[18] Tan Y., Kong C., Yu L., Li P., Chen C., Zheng X., Hertzberg V., Yang C. 4sdrug: Symptom-based set-to-set small and safe drug recommendation. Proceedings of the 28th ACM SIGKDD Conference on Knowledge Discovery and Data Mining.

[19] Ye Q., Hsieh C., Yang Z., Kang Y., Chen J., Cao D., He S., Hou T. (2021). A unified drug–target interaction prediction framework based on knowledge graph and recommendation system. Nat. Commun..

[20] Charoo N., Dharani S., Khan M., Rahman Z. (2023). Nitroso impurities in drug products: An overview of risk assessment, regulatory milieu, and control strategy. AAPS PharmSciTech.

[21] Lee J., Beers J., Geffert R., Jackson K. (2024). A Review of CYP-Mediated Drug Interactions: Mechanisms and In Vitro Drug-Drug Interaction Assessment. Biomolecules.

[22] Marzolini C., Kuritzkes D., Marra F., Boyle A., Gibbons S., Flexner C., Pozniak A., Boffito M., Waters L., Burger D. (2022). Recommendations for the management of drug–drug interactions between the COVID-19 antiviral nirmatrelvir/ritonavir (Paxlovid) and comedications. Clin. Pharmacol. Ther..

[23] Gunn R., Aston E., Metrik J. (2022). Patterns of cannabis and alcohol co-use: Substitution versus complementary effects. Alcohol. Res. Curr. Rev..

[24] Johnson A., Pollard T., Shen L., Lehman L., Feng M., Ghassemi M., Moody B., Szolovits P., Celi L., Mark R. (2016). MIMIC-III, a freely accessible critical care database. Sci. Data.

[25] Clough E., Barrett T. (2016). The gene expression omnibus database. Statistical Genomics: Methods and Protocols.

[26] Knox C., Wilson M., Klinger C., Franklin M., Oler E., Wilson A., Pon A., Cox J., Chin N.L., Strawbridge S. (2024). DrugBank 6.0: The DrugBank knowledgebase for 2024. Nucleic Acids Res..

[27] Xiong G., Yang Z., Yi J., Wang N., Wang L., Zhu H., Wu C., Lu A., Chen X., Liu S. (2022). DDInter: An online drug–drug interaction database towards improving clinical decision-making and patient safety. Nucleic Acids Res..

[28] Johnson A.E.W., Bulgarelli L., Shen L., Gayles A., Shammout A., Horng S., Pollard T.J., Hao S., Moody B., Gow B. (2023). MIMIC-IV, a freely accessible electronic health record dataset. Sci. Data.

[29] Hodson T. (2022). Root mean square error (RMSE) or mean absolute error (MAE): When to use them or not. Geosci. Model. Dev. Discuss..

[30] Pantsar T., Poso A. (2018). Binding affinity via docking: Fact and fiction. Molecules.

[31] Zhou T., Zhang Z., Wang L., Wang X. 3D Molecular Docking Study of Drug-Drug Interactions Between Antidepressants and Immunosuppressive Drugs. Proceedings of the 2025 28th International Conference on Computer Supported Cooperative Work in Design (CSCWD).

